# Outbreaks of enterovirus D68 in Malaysia: genetic relatedness to the recent US outbreak strains

**DOI:** 10.1038/emi.2015.47

**Published:** 2015-08-05

**Authors:** Kim Tien Ng, Xiang Yong Oong, Yong Kek Pang, Nik Sherina Hanafi, Adeeba Kamarulzaman, Kok Keng Tee

**Affiliations:** 1 Department of Medicine, Faculty of Medicine, University of Malaya, 50603, Kuala Lumpur, Malaysia; 2 Department of Primary Care Medicine, Faculty of Medicine, University of Malaya, 50603, Kuala Lumpur, Malaysia

## 

**Dear Editor,**

Enterovirus D68 (EV-D68) is a member of the *Picornaviridae* family, that has been detected sporadically among patients with respiratory infections.^1,2^ However, on August 2014, the US Centers for Disease Control and Prevention (CDC) (Atlanta, GA, USA) was notified by hospitals in Missouri and Illinois of an increase in hospitalized patients presenting with severe respiratory illnesses associated with EV-D68.^3^ Since then, EV-D68 outbreaks have been reported in various states in the USA, and as of January 15, 2015 a total of 1153 laboratory-confirmed cases of EV-D68 have been detected in 49 states and the District of Columbia (http://www.cdc.gov/non-polio-enterovirus/outbreaks/EV-D68-outbreaks.html). Early reports of the whole-genome analysis of EV-D68 strains isolated from Missouri, Illinois, and Kentucky indicated that these strains were probably related to strains detected previously in Asia.^4,5,6^

Despite its epidemiological and clinical impact, information on EV-D68 circulating in tropical countries remains limited. With approval from the University Malaya Medical Centre Medical Ethics Committee, a total of 3935 consenting outpatients who presented with symptoms of acute upper respiratory tract infections were recruited at the Primary Care Clinics at the University Malaya Medical Centre, Kuala Lumpur, Malaysia between March 2012 and May 2014. Respiratory specimens in the form of nasopharyngeal swabs were collected daily. Symptom types and severity were assessed based on criteria described previously.^7^ Each symptom, namely sneezing, nasal discharge, nasal obstruction, headache, sore throat, hoarseness of voice, muscle ache and cough, were graded as absent, mild, moderate, or severe. Total nucleic acids were extracted using the NucliSENS easyMAG automated nucleic acid extraction system (bioMérieux, Marcy I'Etoile, France)^8^ as described in the manufacturer's protocol. The specimens were then screened for viral pathogens using the xTAG Respiratory Viral Panel (RVP) *FAST* Assay (Abbott Molecular, Toronto, Canada) and analyzed using the Luminex's proprietary Universal Tag sorting system on the Luminex 200 IS platform (Luminex Corp., Austin, Texas, USA)^9^ The respiratory virus panel includes influenza A virus, influenza B virus, respiratory syncytial virus, human coronaviruses (OC43, 229E, NL63, and HKU1), parainfluenza viruses (type 1–4), human metapneumovirus, human bocavirus, adenovirus, human rhinovirus, and enteroviruses. Correlation of EV-D68 infections with meteorological parameters such as ground temperature (°C), relative humidity (%), number of rain days, and the amount of rainfall (mm) collected from the nearest meteorological station (latitude: 3°06′N, longitude: 101°39′E) was analyzed using Pearson's correlation. Specimens positive for enteroviruses were further confirmed using standard molecular approaches that involved amplification and sequencing of the human enterovirus *VP4/VP2* gene using primers described previously.^10^ PCR amplification of the capsid (P1) region (2480 bp) of EV-D68 was performed when *VP4/VP2* gene was identified as EV-D68. A combination of previously published primers, namely 9895-forward, 9565-reverse,^10^ 5′fwd1, 5′rev1, 5′fwd2, 5′rev2,^11^ and newly designed primers were used to amplify the P1 region by forming several overlapping fragments. These newly designed primers are EV68P1.5F1 (5′-TCA AAA TTY ACT GAA CCA GT-3′), EV68P1.5R1 (5′-GTT GCC ATG AAG CTV CCA CA-3′), EV68P1.5R2 (5′-GAT ATG TTT CCT ACT ARA GT-3′), EV68P1.3F1 (5′-CCA GGG CAR GTC CGY AAC ATG-3′), EV68P1.3R1 (5′-CCA YTT GWA AAA GTT YTT GTC-3′), EV68P1.3F2 (5′-GTG GAR TCA ATG GAG AT-3′), and EV68P1.3R2 (5′-GCT GAT TTA TCA CYG TGC GAG-3′). A total of 128 *VP4/VP2*, 376 *VP1*, and 20 P1 of EV-D68 retrieved from GenBank (accessed on 26 February 2015) were analyzed using neighbor-joining method implemented in MEGA version 6 to deduce the viral phylogenies.^12^ The statistical robustness of the branching orders was evaluated by bootstrap analysis of 1000 replicates.

In this molecular epidemiological surveillance, approximately 51% (2009/3935) of patients had at least one viral pathogen detected by the multiplex assay, of whom 0.6% (12/2009) were infected with EV-D68. These EV-D68 cases were detected in the second half of 2012 (June to December) and between December 2013 and January 2014, of which September 2012 and January 2014 were the peak months of infection. No EV-D68 cases were detected in other months, suggesting transient outbreaks of EV-D68. The 12 patients (five males and seven females) from whom EV-D68 was detected ranged in age from 29 to 78 years old. During recruitment, most of the patients experienced mild sneezing and moderate-to-severe cough. Correlation of EV-D68 infections with meteorological factors was not observed (correlation coefficient <0.3).

Based on previously described EV-D68 classification,^11^ the newly sequenced strains from Malaysia were found within clade A (MY-Cluster-1) and clade B (MY-Cluster-2). Phylogenetic analysis of the P1 region indicated that 91.7% (11/12) of the Malaysian EV-D68 formed clusters, suggesting the transient EV-D68 outbreaks were most likely caused by at least two viral lineages (Figure 1). A sporadic EV-D68 strain (12MYKL1236) that did not cluster with the major lineages was also observed. Of note, MY-Cluster-1 contains EV-D68 mostly sampled in 2012, while MY-Cluster-2 contains EV-D68 sampled in 2013/2014. Such observation suggests an ongoing “clade shift” or lineage replacement of circulating EV-D68 in causing new outbreak, as observed in other enterovirus-associated outbreaks.^13^ Sequence homology comparison based on the P1 region indicated that the Malaysian EV-D68 strains shared 86.6%–87.6% (nucleotide) and 93.4%–93.9% (amino acid) sequence identities with the Fermon strain (AY426531), a prototype strain isolated in the USA in 1962. Phylogenetic tree also showed that the representative EV-D68 sequences sampled from recent outbreaks in the USA were grouped into three distinct lineages. These lineages are designated as outbreak lineage 1 (consists of sequences isolated from Missouri, MO), lineage 2 (Illinois, IL and Kentucky, KY), and lineage 3 (KY) (Figure 1). Interestingly, Malaysian EV-D68 sequences (MY-Cluster-2) was related to outbreak lineage 1 while outbreak lineage 3 (US/KY/14-18953) was clustered with the MY-Cluster-1, supported by strong statistical evidence (bootstrap values of 97% and 100%, respectively) at the internal tree nodes and genetic distance of ≤0.015. Likewise, outbreak lineage 1 was closely related to the EV-D68 sequences (CU134 and CU171) reported recently in Thailand. All members of MY-Cluster-2 and outbreak lineage 1 exhibited unique amino acid substitution at two residue positions (S647A and T650A), while members of MY-Cluster-1 and outbreak lineage 3 sequences displayed unique amino acid substitutions at four residue positions (N554E, G558E, F655Y, and I739V) relative to the Fermon strain (Figure 1). These “clade-defining” substitutions differentiate outbreak lineage 3 from other outbreak and non-outbreak lineages. A unique substitution T650A discriminated outbreak lineage 1 from outbreak lineage 2, further suggesting that the former is indeed closely related to the Thai strains.^5^ Contribution of these amino acid substitutions on virus pathogenesis however remains unclear. Although the amino acid substitutions are located in the putative immunogenic BC and DE loops, experiments in susceptible cell systems are needed to prove the importance of such substitutions.^14^

The divergence dates of the Malaysian outbreak and other major lineages were estimated by the Bayesian coalescent-based relaxed molecular clock model with constant population size,^11^ implemented in the BEAST software.^15^ Based on the complete *VP1* gene, it was estimated that the Malaysian EV-D68 strains might have emerged around March 2010 (MY-Cluster-1) and November 2012 (MY-Cluster-2) but continued to circulate at a low level. Although recent EV-D68 outbreak in the USA was first observed around mid-August 2014, genealogical estimates indicated that these outbreak lineages might have emerged in May 2013 (outbreak lineage 1), November 2013 (outbreak lineage 2), and November 2011 (outbreak lineage 3), indicating the quiescent persistence of EV-D68 in human population prior to causing large outbreaks.

Our data suggest that the recent EV-D68 strains associated with unprecedented severe respiratory outbreaks in the USA in 2014 were probably descended from the recent EV-D68 lineages circulating in Thailand and Malaysia. However such observation remains presumptuous largely because limited EV-D68 sequence data originating from the USA (due to inadequate surveillance) are available for genealogical analysis. Given the close relationship of the Southeast Asian isolates with the US strains from recent outbreak, a more active enterovirus surveillance should be in place to monitor risks of impending outbreak.

## Figures and Tables

**Figure 1:**
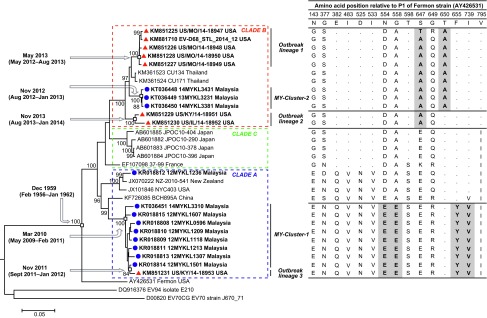
Sequence comparison of EV-D68 isolated from Kuala Lumpur, Malaysia with strains from the recent US outbreak and other global strains retrieved from GenBank. Phylogenetic reconstruction based on EV-D68 capsid (P1) region (2480 bp) showed the relationship of Malaysian EV-D68 (blue circles) with the recent US outbreak strain (red triangles). The robustness of the branching orders was evaluated by bootstrap analysis of 1000 replicates. Divergence time at the internal tree nodes was estimated using the Bayesian Evolutionary Analysis by Sampling Trees (BEAST) software. Amino acid alignment of the P1 region indicates that the MY-Cluster-2 and outbreak lineage 1 exhibited unique substitutions at S647A and T650A while MY-Cluster-1 and US outbreak strain (US/KY/14-18953) displayed unique substitutions at four amino acid residue positions (N554E, G558E, F655Y, and I739V) relative to the prototype Fermon strain.
